# Leadership and capacity building in international chiropractic research: introducing the chiropractic academy for research leadership (CARL)

**DOI:** 10.1186/s12998-018-0173-3

**Published:** 2018-02-06

**Authors:** Jon Adams, Greg Kawchuk, Alexander Breen, Diana De Carvalho, Andreas Eklund, Matthew Fernandez, Martha Funabashi, Michelle M. Holmes, Melker S. Johansson, Katie de Luca, Craig Moore, Isabelle Pagé, Katherine A. Pohlman, Michael S. Swain, Arnold Y. L. Wong, Jan Hartvigsen

**Affiliations:** 1 Faculty of Health, University of Technology Sydney, Sydney, Australia; 2 Faculty of Rehabilitation Medicine, University of Alberts, Edmonton, Canada; 3 AECC University College, Bournemouth, UK; 4 Faculty of Medicine, Memorial University of Newfoundland, NL, St. John, ’s Canada; 5 Institute of Environmental Medicine, Karolinska Institutet, Stockholm, Sweden; 6 Private practice of chiropractic, Drummoyne, NSW Australia; 7 Department of Psychology, University of Southampton, Southhampton, UK; 8 Department of Sports Science and Clinical Biomechanics, University of Southern Denmark, Campusvej 55, 5230 Odense M, Denmark; 9 National Research Centre for the Working Environment, Copenhagen, Denmark; 10 Private practice of chiropractic, South West Rocks, South West Rocks, NSW Australia; 11 Department of Anatomy, Université du Québec à Trois-Rivières, Trois-Rivières, Québec, Canada; 12 Research Institute, Parker University, Dallas, TX USA; 13 Department of Chiropractic, Faculty of Science and Engineering, Macquaire University, Sydney, Australia; 14 Department of Rehabilitation Sciences, The Hong Kong Polytechnic University, Kowloon, Hong Kong; 15 Nordic Institute of Chiropractic and Clinical Biomechanics, University of Southern Denmark, Odense, Denmark

**Keywords:** Chiropractic, Leadership, Research, Evidence

## Abstract

In an evidence-based health care environment, healthcare professions require a sustainable research culture to remain relevant. At present however, there is not a mature research culture across the chiropractic profession largely due to deficiencies in research capacity and leadership, which may be caused by a lack of chiropractic teaching programs in major universities. As a response to this challenge the Chiropractic Academy for Research Leadership, CARL, was created with the aim of develop a global network of successful early-career chiropractic researchers under the mentorship of three successful senior academics from Australia, Canada, and Denmark. The program centres upon an annual week-long program residential that rotates continental locations over the first three-year cycle and between residentials the CARL fellows work on self-initiated research and leadership initiatives. Through a competivite application process, the first cohort was selected and consists of 13 early career researchers from five professions in seven countries who represent diverse areas of interests of high relevance for chiropractic. The first residential was held in Odense, Denmark, with the second being planned in April 2018 in Edmonton, Canada, and the final residential to be held in Sydney, Australia in 2019.

## Background

Health care professions require a sustainable research culture to underpin practice and justify effective, safe coordination and integration of care within the wider health care system [1]. Despite numerous calls for more research [2–7], at present, a mature research culture across the chiropractic profession remains elusive, largely due to deficiencies in research capacity and leadership [8]. The interrelated tasks of building research capacity and facilitating research leadership in chiropractic are especially pertinent given some parts of chiropractic’s contemporary focus on evidence-based health care [9]. Futhermore, it is pertinent as a core foundation to produce a sound evidence base relevant to appropriate practice and policy decision-making [10]. Building research capacity and facilitating research leadership can also have significant positive effects to advance a broader leadership capability and a wider professional development of chiropractic beyond the research realm [8].

Given the need to develop research capacity and leadership within chiropractic, a group of senior health researchers (Adams, Hartvigsen and Kawchuk) having research interests of relevance to chiropractic, have planned and founded the world-first international chiropractic research leadership initiative – the Chiropractic Academy of Research Leadership (CARL).

## The aims of the CARL program

The CARL program is driven by a number of distinct but interrelated aims. While there are undoubtedly significant pockets of chiropractic research activity dotted around the world, a challenge for many in this field has been a lack of global networks and infrastructure. The primary aim of the CARL program is therefore to address this gap through mentoring of early career researchers into a cohesive cohort. As such, this program provides an opportunity for geographically disparate researchers to share interests and insights and ultimately develop longstanding, hopefully lifelong, friendships and collaborations helping *to develop a critical mass of successful early-career chiropractic researchers on the international stage*.

Another aim of the CARL program is to *encourage multi-disciplinary perspectives and cooperation in chiropractic research* – not only across different disciplinary trainings and expertise but also across the researcher/practitioner fields and chiropractic/non-chiropractic divide where those exist. As with many budding research fields, chiropractic academia can in some cases be a highly competitive environment and CARL encourages appointed Fellows to co-ordinate and collaborate their efforts wherever possible.

The program also aims *to provide much-needed ‘time-out’ from the day-to-day pressure of work environments allowing space and time to reflect and consider longer-term, deeper issues* around both individual career development and strategic blue-sky planning for the profession more broadly.

CARL further aims to *develop confidence amongst these future leaders in the profession*. It is important for a research culture that researchers are confident both in their own location in the research and academic world as well as in their discipline and focus or topic. This can be as simple as reaffirming that chiropractic is indeed an important field amongst many others in the health system worthy of its own independent and integrative examination and assessment.

The CARL program additionally aims *to provide direct mentorship, support and advice to a cohort of CARL Fellows regarding their own research focus, strategic development and career pathway*. There are countless potential opportunities and hazards in advancing any academic career and CARL encourages Fellows to identify and explore these in a ‘safe’ place away from what can sometimes be the competitively-driven environment of a home institution. Peer-support is a core ingredient in advancing this aim. Importantly, 50% of the program’s contact time focuses on providing leadership skills and identifying leadership opportunities within the profession. This critical feature recognizes that a relatively small percentage of early career health researchers remain within academia in the face of high rejection rates and an increasingly competitive environment [11–13]. The CARL program not only helps strengthen future researchers, but also aims to build a cohort of high-functioning leaders who have been exposed to other career opportunities within chiropractic.

Finally, CARL aims *to promote and produce a prolific range of tangible research outputs and products* helping to bolster and coordinate efforts to build an evidence base to inform practitioners, patients, policymakers and other relevant stakeholders. The focus is upon not only development of new collaborative projects and project funding but also dissemination via peer-review publications, professional and research conferences and community-based engagement.

## Core principles of CARL

A number of core features are arguably important to develop sustainable, effective health research capacity and leadership. Foremost, the development should acknowledge and reflect issues and topics of relevance to practitioners and their patients. Unfortunately, health research to date, beyond and within chiropractic, has illustrated a disconnect between research and clinical practice [14–16], and it is important that future chiropractic enquiry not be defined by these same challenges [17].

Another core feature of CARL will be the camaraderie of high achieving, like-minded individuals placed together within a supportive and productive environment. It is known that feelings of isolation, the lack of professional support and/or recognition from within the chiropractic profession are substantial challenges faced by higher-degree chiropractic research students and can be a major barrier to research retention [18]. Moreover, the challenges of career advancement within a competitive climate, overcoming disappointment and managing researcher ‘burnout’ are often either dealt with in isolation or not discussed. While most chiropractors opt for clinical practice, providing mentorship in a safe, prosperous environment for early career researchers will enhance their research capability, leadership and career development.

Another closely related core feature of CARL is to encourage and reflect both a broad range of perspectives beyond the chiropractic profession and the investigation of topics that stretch beyond those exclusively of interest to chiropractic. As such, CARL will include collaborations and an interface with experts from across the wider academy (e.g. clinical researchers, trial methodologists, public health and health service researchers, social scientists and health economists amongst many others) not only enriching empirical investigation but also safeguarding research from adopting predominantly or exclusively ‘chiropractic-centric’ perspectives.

Multi-discplinary collaboration is also essential to ensuring chiropractic is subjected to the same rigorous critical methodological testing and evaluation as other areas of health practice – a perspective that advances the entire health care field within and beyond conventional medical practice. Such a stance helps maintain internal and external validity and provides the potential for a legitimate evidence base essential if chiropractic care is to advance within the wider health system.

Finally, it is important to develop chiropractic research focus beyond the short-term [19]. CARL will develop a road-map and the infrastructure to facilitate strategic research direction, growth and sustainability for future investigators and investigations will encourage a co-ordinated, big-picture approach – issues of crucial significance given the relative infancy of the chiropractic research culture in many jurisdictions around the world.

## The evolution and methodology of CARL

CARL was initially built upon the personal experience of Distinguished Professor Jon Adams as an appointed Senior Fellow (2009) on the Oxford International Primary Care Research Leadership Program at the Nuffield Department of Primary Care Health Sciences, University of Oxford (www.phc.ox.ac.uk). In late 2015 Distinguished Professor Adams, Professor Hartvigsen and Professor Kawchuk collaborated on the idea of a similar international research leadership program around chiropractic and the concept of the CARL Program was founded. The CARL Program identifies and mentors talented, promising early career researchers over an initial three-year period with future program years planned.

The design and content of the CARL Program was in part informed by the list of aims outlined earlier as well as the foundational features of the Oxford International Primary Care Research Leadership Program. As a result, the program centres upon an annual week-long program residential that rotates continental locations over the first three-year cycle. Each residential consists of an intense week of activities whereby Fellows receive individual mentorship, problem-solving, career development, insights into academic and research management and presentations and workshops from invited international senior academics. Alongside these structured sessions and activities, Fellows are also allocated time to explore areas of mutual interest for collaboration and partnership. In addition, an essential feature of the program and residential is to facilitate and encourage ‘unstructured’, organic interactions and conversations between Fellows who, in many cases, may not have necessarily had an opportunity to investigate and explore collaborative opportunities with each other outside the CARL Program. Between residentials, the Fellows work on research and leadership projects using electronic and internet platforms for project management and communication. Importantly, while the mentors inspire activities, the drive of the program quickly becomes the charge of the Fellows who shape and mold future directions and projects, with mentor support, as their confidence and experience grows.

## The first CARL cohort

The initial worldwide call for CARL Fellowship submissions attracted over 30 full applications from international candidates. Following extensive peer-review by the founders and a round of short-listed candidate interviews, the first cohort of the CARL program has now been selected and consists of 13 early career leaders from 5 professions in 7 countries who represent diverse areas of interests of high relevance for chiropractic (Table 1). Six are women, six are PhD students, four are Postdoctoral Fellowss, three are Assistant Professors, and four work as part time clinicians in addition to their research activities. With regards to their motivations for joining CARL, all Fellows have reported the need for and benefit of being involved in an international network helping to strengthen their research and leadership careers, the importance of receiving explicit mentorship and developing collaborations and friendships from their program involvement.

**Table 1:** Descriptive details about CARL fellows and own statements about reasons for joining CARL

Country, sex, academic degrees	Current positions	Why did you join CARL?	What do you expect to get out of CARL?	How might CARL benefit chiropractic?
Australia, ♂, chiropractor, MSc	PhD student Part-time clinician	Want to be part of global collaboration among ECRs to move chiropractic forward	Mentorship and help to participate in research projects	Coordinated international research planning and output is critical to the progress and standing of the chiropractic profession within mainstream health
Sweden, ♂, chiropractor, MSc, PhD	Post Doc Part-time clinician	Want to engage in global collaboration and get exposure to other research groups and professional networks with a common focus on chiropractic/manual medicine	Get exposure to new methods and applications, expand knowledge and skills. Increased publication output and research experience	Help build research capacity in Chiropractic. Increase the output and quality of research relevant to the Chiropractic profession
Canada, ♀, chiropractor, MSc	PhD student Part time clinician	Huge opportunity for PhD student who wants to continue in the field of chiropractic research and believes international collaboration is of prime importance	Become part of strong international network. Develop research skills and independence	CARL allows for the development of a strong network of researchers around the word that have the capacity to collaborate and support each other
Australia, ♀, chiropractor, MSc, PhD	Post Doc Part-time clinician	Want to become part of international network and learn from key international academics in supportive environment	Boost in research career and high-quality research output. Broaden opportunities for collaborating with international researchers	The CARL leadership projects will benefit all chiropractors, with CARL fellows dedicated to establishing and endorsing initiatives that will foster growth and increased visibility for the chiropractic profession
UK, ♀, Natural therapeutics, MSc	PhD student Lecturer	Want to be able to co-ordinate my research with researchers internationally in order to advance the field	Collaborate with other fellows to identify areas of research priority for the profession. Having mentorship	Through the CARL program, the CARL fellows can plan research projects to have a successful impact in practice and contribute to the international research agenda within chiropractic and the future of the profession
Canada, ♀, chiropractor, MSc, PhD	Assistant Professor	I joined CARL to develop and expand my international network for collaborations, for early career mentorship and to ensure my research continues to have an impact in the field of chiropractic	I expect to learn from and work with my colleagues in order to become a better researcher and leader in the chiropractic profession	CARL can elevate the profession in terms of research productivity and impact and connect future leaders that can motivate and inspire chiropractic practitioners worldwide to integrate evidence to the benefit of their patients
Australia, ♂, chiropractor, PhD	Clinician Lecturer	To work with like-minded early career researchers, continuing my education in research, and the opportunity to be mentored by leaders in the field	Leadership skills, publication output, friendship and being part of an up-and-coming early research culture within the profession	Change the landscape, where private practice providers can appreciate and further utilize high-quality research, impacting their practice in an evidence-based manner – establishing chiropractic cultural authority
Canada, ♀, physical therapist, MSc, PhD	Post Doc Research Associate	To learn from successful mentors as well as to build strong collaborations to assist with increasing the number of publications and planning/conducting future high-quality studies. For my surprise, CARL was much more than that	Learn from mentors’ experience get their guidance. To build strong relationships with the other CARL fellows, which will lead to strong collaborations that will significantly contribute to the advancement of chiropractic and SMT research	Bringing the young career researchers that have the greatest potential of becoming successful investigators in chiropractic together, giving them the opportunity to bond, have ideas, work together and support each other is a brilliant idea that paves the way for fruitful collaborations and high-quality studies that will greatly advance the evidence regarding the chiropractic profession
Australia, ♂, chiropractor	PhD student Lecturer	I joined CARL to meet people who share my passion for developing the scholarship of chiropractic, and to work collaboratively with them into the future for the befit of the community	An international network of career long collaborators that will answer important research questions and expand the relevant knowledge base and international leadership	To exist, a profession must have a knowledge base, quality education, and ongoing expansion of that knowledge base through postgraduate scholarly activity and research. CARL serves a necessary contribution from a profession to the betterment of society
UK, ♂, MSc, PhD	Post Doc	I applied to join CARL to put my work into the context of other chiropractic researchers. My long-term aim is to understand dynamic individualized biomechanical factors in low back pain and help to explain the effects of spinal manipulation.	The CARL program has helped help me to gain a deeper appreciation of research areas beyond my own focus in biomechanics. In return, it is my hope that my own expertise in biomechanics might help the other CARL fellows	A community of early career researchers from across the board of chiropractic research based all over the world can do more for the scientific progress of the profession than individual experts aligned to their more disparate and isolated topics. I am confident that this program can promote a synergy between the biological, physiological and social aspects of the biopsychosocial model
Denmark, ♂, chiropractor, MSc	PhD student	CARL came across as a great opportunity to meet like-minded research colleagues and friends, for sharing experiences and improving research-related skills	CARL constitutes an excellent Forum, just like a greenhouse, where we can discuss issues and barriers related to research and career development, learn and be inspired; collaborate, and ultimately flourish as health care researchers	CARL will strengthen the profession by facilitating international research collaborations, fostering future leaders and thereby further position the chiropractic profession as an important contributor to health care and health care research globally
USA, ♀, chiropractor, MSc	PhD student Assistant Professor	CARL opens up the opportunity to create the most impactful and innovative chiropractic research, which is my desire for the special pediatric, pregnant, and post-partum population	As the solo chiropractic clinical scientist, CARL is for mentorship in research capacity building. With this network, chiropractic research could become united, cohesive, and impactful	CARL just fast-tracked chiropractic research by networking early career scientists and providing the platform for collaboration. This effort will undoubtingly impact the current evidence-based chiropractic literature gap and most importantly the management and care of chiropractic patients
Hong Kong, physical therapist, MSc, PhD	Assistant Professor	As an early career researcher with a passion to conduct high quality research, I want to have some mentors and peers to guide and support my research journey	To broaden my research skills and network through collaboration. I am determined to be a prominent research leader and conduct impactful chiropractic research and nurture new research leaders	Given the complexity of spinal problems, it is important to coordinate personal resources from different disciplines. This program can empower the next generation of chiropractic research leaders through research collaborations, grant writing and knowledge translation

♀ = Female

♂ = Male

## Discussion: progress and successes of CARL program to date

Health professions operating beyond the conventional medical profession and curriculum are increasingly realising that they need to increasingly engage with the larger societal agenda of evidence-based practice [20]. Chiropractic is among the largest health care professions in the world beyond conventional medicine. While there are existing research groups in chiropractic who are productive by any standard, global research activities in chiropractic remain modest. In response to these circumstances, the Chiropractic Academy for Research Leadership (CARL) Program was founded in 2016. This model will provide the longevity and impact to deliver one platform and growth strategy, which is required to develop a mature, sustainable international chiropractic research and leadership culture. With the first cohort of Fellows appointed in late 2016, the inaugural CARL residential was held at the University of Southern Denmark campus in Odense, Denmark in April 2017. The 5-day program included guest lectures from successful young researchers, experienced musculoskeletal research leaders, experts on management and leadership as well as many workshops and social activities. From these interactions and scheduled work time, the primary outcome for the first CARL residential was produced; a catalogue of collaborative research and leadership opportunities for the Fellows to pursue over the coming year. Specifically, the catalogue currently consists of numerous identified academic and leadership projects. In the six months since the first residential in Denmark, CARL Fellows have submitted a variety of papers to peer-reviewed journals and have completed numerous leadership activities. A fellow-driven newsletter will be released twice a year, with the first and subsequent newsletter available at https://chiroresearchfellows.wordpress.com/.

The next residential is now being planned for Edmonton, Canada in April 2018 and will bring the Fellows together to seed the Program catalogue with new ideas and review progress as well as provide additional advanced training in research productivity and leadership skills. The following week, the CARL Fellows will also take part and showcase their abilities at the Canadian Chiropractic Association Convention in Calgary.

## Conclusion

The long-term aim of CARL is to develop the essential leadership skills and experiences to take on senior CARL mentorship appointments and help secure the successful mentorship of a subsequent early-career researcher cohort of Fellows. The initial cohort of 13 appointed CARL Fellows has shown excellent promise and produced numerous outputs in the first six months since the program launch and it is envisaged that this first cohort can be supported on a more ongoing basis.

## Abbreviations

CARL: Chiropractic Academy of Research Leadership
